# An Improved DDPG and Its Application in Spacecraft Fault Knowledge Graph

**DOI:** 10.3390/s23031223

**Published:** 2023-01-20

**Authors:** Xiaoyu Xing, Shuyi Wang, Wenjing Liu

**Affiliations:** 1 Beijing Institute of Control Engineering, Beijing 100190, China; 2 Science and Technology on Space Intelligent Control Laboratory, Beijing 100190, China

**Keywords:** knowledge graph, relational reasoning, representation learning, deep reinforcement learning

## Abstract

We construct a spacecraft performance-fault relationship graph of the control system, which can help space robots locate and repair spacecraft faults quickly. In order to improve the performance-fault relationship graph, we improve the Deep Deterministic Policy Gradient (DDPG) algorithm, and propose a relationship prediction method that combines representation learning reasoning with deep reinforcement learning reasoning. We take the spacecraft performance-fault relationship graph as the agent learning environment and adopt reinforcement learning to realize the optimal interaction between the agent and the environment. Meanwhile, our model uses a deep neural network to construct a complex value function and strategy function, which makes the agent have excellent perceptual decision-making ability and accurate value judgment ability. We evaluate our model on a performance-fault relationship graph of the control system. The experimental results show that our model has high prediction speed and accuracy, which can completely infer the optimal relationship path between entities to complete the spacecraft performance-fault relationship graph.

## 1. Introduction

In recent years, space robots used for spacecraft fault maintenance have been widely considered. They need accurate and fast fault location ability, which is one of the difficulties in application. At present, the mapping relationship between the performance and fault of a spacecraft control system is generally expressed in the form of failure mode and effects analysis (FMEA) or a fault tree. When the spacecraft control system is abnormal, the ground experts can locate the fault source by manual inquiry, so it is difficult to make real-time diagnosis, accurately locate complex faults, and visualize fault diagnosis. Knowledge graph [1,2,3,4,5,6,7,8,9], as an effective tool to describe massive knowledge, knowledge attributes, and knowledge relationships, provides a new means for fault diagnosis. We can manually or automatically construct the knowledge graph of the corresponding relationship between the performance and fault of the spacecraft control system by combining various model knowledge, expert knowledge, and data, which we call the spacecraft performance-fault relationship graph. However, there is no systematic and comprehensive spacecraft fault knowledge base in the aerospace field, so it is difficult to construct a large-scale spacecraft performance-fault relationship graph for spacecraft fault diagnosis. Taking the spacecraft control system as an example, its working environment is harsh. Because of its closed-loop characteristics, there are few fault samples accumulated during long-term in-orbit operation, and the fault mechanism cannot be traversed. Meanwhile, its structure is complex, and the components are closely related. Therefore, it is difficult to establish an accurate and complete performance-fault relationship graph of a spacecraft control system, which affects the accuracy of fault diagnosis results. It is necessary to use relational reasoning to predict the relationship and complete the relationship.

The commonly used relationship reasoning methods at present include representation learning reasoning, path reasoning, reinforcement learning reasoning, and graph convolution network reasoning. However, the spacecraft performance-fault relationship graph has complex relationships, various entities, and rich physical meanings. These methods are difficult to directly apply to a spacecraft performance-fault relationship graph.

The TransE [10] model is a classic model for learning reasoning. It maps entities and relationships into a continuous vector space, and calculates the entity vectors to predict the relationships. However, this method is only suitable for dealing with the one-to-one correspondence of entities and is not suitable for spacecraft performance-fault relationship graphs with complex and diverse relationships. A typical path reasoning model is the Path Ranking Algorithm (PRA) [11], which extracts path feature values from the knowledge graph, and makes relationship judgment according to path features. COR-PRA (Constant and Reversed Path Ranking Algorithm) [12] adds a constant path search mechanism on the basis of PRA, which makes the relationship reasoning more comprehensive. However, path reasoning pays too much attention to the graph structure information, ignoring the semantic attributes of entities and relationships, and the reasoning accuracy is not high. Reference [13] first applied reinforcement learning to relational reasoning and put forward a reinforcement learning framework DeepPath for learning multi-hop relational paths. Based on this, reference [14] added long short-term networks and a graphic attention mechanism as memory components. Reference [15] presented agent MINERVA, which has the built-in flexibility to take paths of variable length and learns to perform query answering by walking on a knowledge graph conditioned on an input query. References [16,17,18,19] improved reward functions and strategies for different datasets based on reinforcement learning reasoning. However, reinforcement learning reasoning requires setting a high reward function and value function. For the spacecraft performance-fault relationship graph with various states and complex physical meanings, it is difficult to set an accurate reward function and value function to achieve a good path finding effect. The graph convolution network [19,20,21,22,23,24,25,26,27,28,29] directly aggregates the adjacent nodes of the graph, which can better reflect the structural relationship of graph data and is more suitable for processing directed graph and large graph data. However, its model is complex and lacks interpretability, so it is not suitable for the complicated spacecraft performance-fault relationship graph.

To solve the above problems, we improve the DDPG algorithm [30] by combining representation learning reasoning with deep reinforcement learning reasoning. We study a Semantic relation and Position Deep Deterministic Policy Gradient reasoning model (SPDDPG) and apply it to the spacecraft performance-fault relationship graph to realize relational reasoning. First, this model extracts the semantic and location features of the embedded vectors of entities and relations. The high-dimensional semantic embedded vectors of entities and relations are obtained by using the TransE model. However, its excessively high dimension and scattered features will affect the reasoning accuracy. To solve this problem, Principal Component Analysis (PCA) [31] is adopted to reduce its dimension to obtain the key semantic feature vectors. Meanwhile, according to the position of the entity in the spacecraft performance-fault relationship graph, the position vector is obtained by Boolean vector conversion. Then, we splice the semantic vector and the position vector as the embedded vector of the entity and input it into DDPG for processing. DDPG takes the spacecraft performance-fault relationship graph as the environment, in which we set different corresponding states and rewards for complex entity types in the spacecraft performance-fault relationship graph. The strategy network of DDPG outputs the optimal action vector according to the embedded vector of the current state, and the value network calculates the action value according to the state vector and the action vector, updating the network parameters in reverse. Both the strategy network and value network are deep neural network models, which can fit complex strategy and value functions. Thus, they are suitable for the spacecraft performance-fault relationship graph with intricate relationships and complex physical meanings.

In short, this model can achieve accurate reasoning of the spacecraft performance-fault relationship graph due to two innovations: 1. It extracts the semantic information and position information from the spacecraft performance-fault relationship graph. 2. It has a reasonable network and state setting to realize fast optimal action prediction.

## 2. Materials and Methods

### 2.1. Fault Diagnosis Process Based on Spacecraft Performance-Fault Relationship Graph

The fault diagnosis process based on the spacecraft performance-fault relationship graph is divided into two parts. First, data processing and feature extraction are carried out on spacecraft on-orbit data, and the obtained results are matched with fault symptom entities in the performance-fault relationship graph. The fault symptom entity corresponding to the current on-orbit data is then determined. Second, in the performance-fault relationship graph, reasoning is started from the failure symptom, and the failure cause entity or failure mode entity corresponding to the current failure symptom is obtained through an entity-relationship-entity triple search. There may be many fault causes or modes, so the final fault diagnosis result should be determined by information fusion of fault occurrence probability and expert experience. As a tool of fault diagnosis, the spacecraft performance-fault relationship graph must be complete and accurate. We propose a relational reasoning model SPDDPG for the performance-fault relationship graph and use it to improve the performance-fault relationship graph, which provides reliable and effective support for subsequent fault diagnosis.

### 2.2. SPDDPG Model and Process

**Figure 1:**
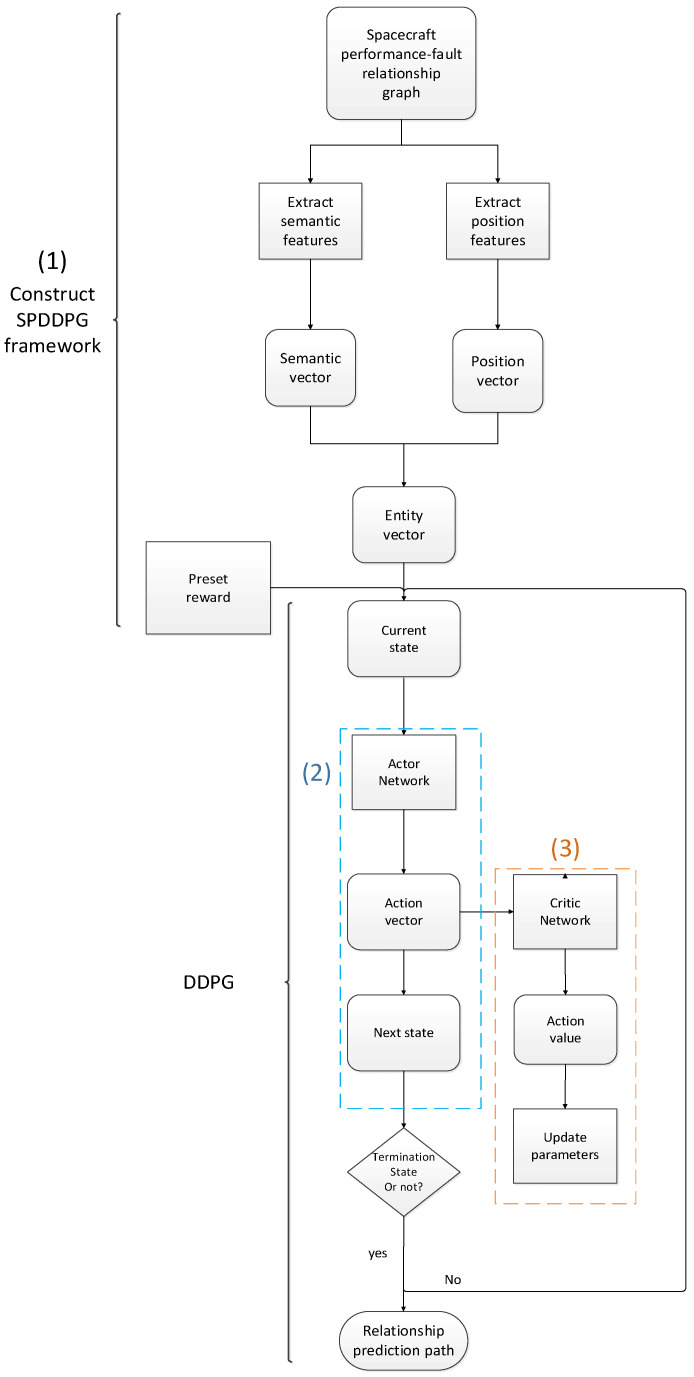
The reasoning process of SPDDPG.

The steps of relationship reasoning with the SPDDPG model are as follows: 1. Build the SPDDPG framework based on the spacecraft performance-fault relationship graph and transform the components in the relationship graph into the basic elements of the SPDDPG framework. 2. Take the entity in the performance-fault relationship graph as the current state and select the optimal action by using the strategy network, which we call the Actor Network, to obtain the entity corresponding to the next state. 3. Use the value network, which we call the Critic Network, to fit the action value function, and update the parameters of the strategy network and the value network. The reasoning process of this model is shown in Figure 1.

### 2.3. Construct SPDDPG Framework

We use the deep reinforcement learning algorithm for relational reasoning, so we need to construct the reinforcement learning framework first. We configure the basic elements of the framework, including environment, state, action, and reward, according to the performance-fault relationship graph of the spacecraft control system.

**Environment.** We use the performance-fault relationship graph of the spacecraft control system as the environment to interact with the agent. Because the performance-fault relationship graph is difficult to directly calculate and process by agents, we transform it into an *n × n* (where *n* is the number of entities) dimensional environment matrix ***E***. The *u* relationships between entities are arranged in sequence. If there is a relationship *q*∈(0,*u*) between entity *i* and entity *j*, then *E_ij_* is set to *q*; otherwise, it is set to 0. Finally, we obtain environment matrix ***E***, which is convenient for a computer to calculate and process the content of the performance-fault relationship graph.

**State.** The relationship path of performance-fault relationship graph is complex and involves many states, so it is difficult to achieve ideal experimental results only by setting the normal state and the termination state. We divide the states into the normal state, termination state, and imminent termination state. The imminent termination state refers to the first-order adjacent state to the termination state, and when the agent reaches the termination state, it will complete the path finding of relational reasoning. Our innovative setting of the imminent termination state can help the agent to find the path in the direction of the termination state quickly. The entities in the performance-fault relationship graph are taken as the state. However, they need to be converted into a vector form convenient for computer identification and calculation, which is called the state vector in this paper. The state vector includes two parts: semantic features and location features.

The first part is semantic feature extraction. We use the TransE model to process triples in the performance-fault relationship graph, extracting the semantic high-dimensional vectors of entities and relationships. Then, we use PCA to reduce the dimension and extract the principal components of the semantic features of entities and relationships, which we call the semantic vector.

The second part is location feature extraction. We arrange all entities in the order of (*e_1_*, *e_2_*, …*e_n_*), and adopt one-hot coding to obtain the position vectors of all entities. Specifically, we define an *n*-dimensional zero vector as the position vector of every entity. If entity *i* has a relationship with the entity *e_j_*, the *j*th element of the vector is set to 1. The position vector contains the global position characteristics of the entity in the performance-fault relationship graph.

**Action.** In the case of entities as states, the relationships between entities act as the action to connect states. The agent outputs the predicted action vector according to the current state to choose the action to reach the next state. The action vector is represented by the relational semantic vector obtained from the TransE model.

**Reward.** As the basis for judging the current state, a reward should be set artificially according to the path distance and path type between the current state and the termination state. We use the critic network to fit the complex action value function, so the setting of the state reward can follow the simple and effective principle. The normal state occupies most of the path of relational reasoning and should not be set too tendentiously, so it is set to 0. The imminent termination state should guide the agent to the terminal state, so it is set to 1, and the termination state reward is set to 2. The formula is as follows:

$$R_{t}=\left\{\begin{array}{l} 0,\;S_{t}\notin N_{1}\left(END\right)\;and\;S_{t}\neq END\\ 1,\;S_{t}\;\epsilon \;N_{1}\left(END\right)\\ 2,\;S_{t}=END \end{array}\right.\;$$

where *t* is the number of steps of agent path finding, $\;S_{t}$ is the current state, $R_{t}$ is the reward corresponding to the current state, *END* is the end state, and $N_{1}\left(END\right)\;$is the first-order neighborhood of the end state. The unique reward of the terminal state can help the agent approach the terminal state quickly from many complicated paths, and enhance the training effect.

### 2.4. DDPG Model

**Figure 2:**
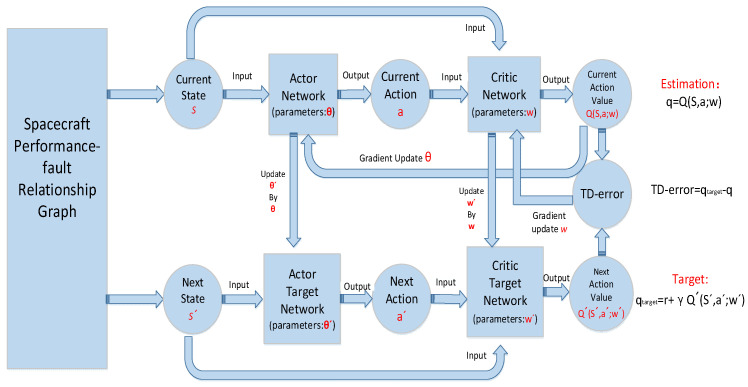
The structure of DDPG model.

The DDPG model consists of two kinds of neural networks, the actor network and critic network. The actor network is responsible for predicting actions according to the state, and the critic network is responsible for predicting action values according to the state and actions. The two types of networks have their own current network and target network. The current network calculates the estimated value according to the current state, while the target network calculates the target value according to the next state. The parameters of the neural network are updated according to the estimated value and the target value. Next, we elaborate action selection based on the actor network and parameter update based on the critic network in detail. Figure 2 shows the structure of the DDPG model.

#### 2.4.1. Action Selection Based on Actor Network

**Figure 3:**
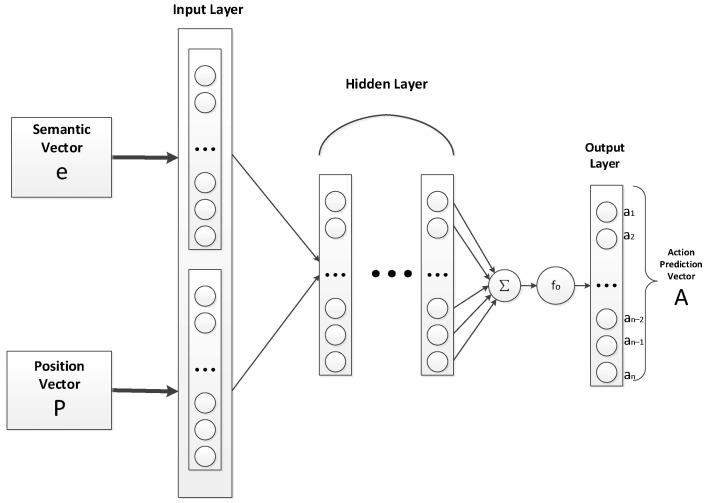
The structure of SPMLP model.

We use the semantic relation and position multilayer perceptron (SPMLP) model as the actor network. We take the state vector as the input of the SPMLP model and output the action prediction vector. The model structure is shown in Figure 3.

We take the entity vector ***X*** and reward *R* corresponding to the current state *S*. The entity vector ***X*** is used as the input vector of the SPMLP input layer. Each element of the entity vector ***X*** is multiplied by the weight *θ*_1_ of the first hidden layer, and summed up. After adding the offset value *b*_1_, the output ***h*_1_** of this layer is obtained through the *sigmoid* activation function *f*_1_, and the formula is as follows:

(1) $$h_{1}=f_{1}(\theta_{1}X+b_{1})$$

Take the output of the first hidden layer as the input of the next hidden layer, and repeat Formula (1) to obtain the output of the last hidden layer as ***h_t_***. The weight is *θ_y_* and the offset is *b_y_*. The activation function *f_o_* of the output layer selects the *softmax* function. The final output action prediction vector ***A*** is as follows:

(2) $$A=f_{o}(\theta_{y}h_{t}+b_{y})$$

The element position of the prediction vector ***A*** corresponds to the relationship in the performance-fault relationship graph. The element *A_i_* with the highest probability in ***A*** is the selected optimal action *a*. Find the next state *S*′ corresponding to the optimal action from the environment matrix ***E***, take *S*′ as the current state, and repeatedly take the above steps until the end state is reached. Finally, the agent outputs the relationship prediction path.

#### 2.4.2. Parameters Updating Based on Value Network

The multilayer perceptron (MLP) model is adopted as the critic network, which takes the action vector and state vector as input and outputs the action value. The hidden layer structure of the model is the same as that of SPMLP, and the calculation process is similar to the previous section. The difference is that the action value output by the value network is a real number, the activation function of the output layer is the *Relu* function, and the network parameter is *w*.

We set-up the actor target network and the critic target network, and the structure is consistent with the actor network and the critic network. The actor target network selects the next optimal action *a*′ according to the next state *S*′, and the network parameter *θ*′ is periodically copied from *θ*. The critic target network calculates the action value *Q*′ (*S*, *a*′, *w*′) of the next state, and the network parameter *w*′ is copied from *w* regularly. Put the {*S*, *A*, *R*, *S*′, *A*′} quintuple of each cycle into the empirical playback set ***D***; take *p* samples *{S_j_, A_j_, R_j_, S_j_′, A_j_′}* from ***D***, where *j =* 0, 1, 2, …. *p*; calculate the current target *Q* value *Q_target_*:

(3) $$Q_{target}=R_{j}+\gamma Q^{\prime }\left({S_{j}}^{\prime },{a_{j}}^{\prime };w^{\prime }\right)\;$$

where *γ* is the discount factor, *γ* ∈ (0, 1), and it is 0.9 in this paper.

Parameters *w* of the current value network are updated by gradient back propagation of the neural network, and MSE (Mean Square Error) is used as the loss function of the value network.

(4) $$\mathrm{MSE}=\frac{1}{p}\overset{p}{\underset{j=1}{\sum }}{\left(Q_{target}-Q\left(S_{j},a_{j};w\right)\right)}^{2}$$

Parameters *θ* of the current strategy network are updated by gradient back propagation of the neural network, and the loss function of the strategy network is:

(5) $$J\left(\theta \right)=-\frac{1}{p}\overset{p}{\underset{j=1}{\sum }}Q\left(S_{j},a_{j};\theta \right)$$

Set the update frequency *c* and soft-update the parameters of the actor target network and critic target network every *c* cycle:

(6) $$w^{\prime }\leftarrow \tau w+\left(1-\tau \right)w^{\prime }$$

(7) $$\theta^{\prime }\leftarrow \tau \theta +\left(1-\tau \right)\theta \text{′}$$

where τ is the renewal coefficient, is generally small, and is 0.1 in this paper.

## 3. Experimental Results and Discussion

### 3.1. Performance-Fault Relationship Graph of Spacecraft Control System

We apply the SPDDPG algorithm to the performance-fault relationship graph of the spacecraft control system constructed by us. First, based on the models of each part of the spacecraft control system, the attitude dynamics equation, and the attitude kinematics equation, we construct the performance-fault relationship graph of the spacecraft control system. Taking the system composed of three orthogonal gyroscopes, rolling axis infrared earth sensors, and three orthogonal momentum wheels as the object, the attitude dynamics equation of the spacecraft is as follows:

(8) $$I_{x}{\dot{\omega }}_{x}+\left(I_{z}-I_{y}\right)\omega_{y}\omega_{z}=-{\dot{h}}_{x}$$

(9) $$I_{y}{\dot{\omega }}_{y}+\left(I_{x}-I_{z}\right)\omega_{x}\omega_{z}=-{\dot{h}}_{y}$$

(10) $$I_{z}{\dot{\omega }}_{z}+\left(I_{y}-I_{x}\right)\omega_{y}\omega_{x}=-{\dot{h}}_{z}$$

where $I_{x}$, $I_{y}$, and $I_{z}$ are the three-axis moments of inertia; $\omega_{x}$, $\omega_{y}$, and $\omega_{z}$ are the components of the spacecraft’s space angular velocity along the main inertia axis; $h_{x}$, $h_{y}$, and $h_{z}$ are the three-axis angular momenta of the momentum wheel.

The attitude kinematics equation of spacecraft is

(11) $$\left\{\begin{array}{c} \omega_{x}=\dot{\varphi }-\omega_{0}\psi \\ \omega_{y}=\dot{\theta }-\omega_{0}\\ \omega_{z}=\dot{\psi }+\omega_{0}\varphi \end{array}\right.$$

where *φ*, *θ*, and *ψ* are Euler angles, and $\omega_{0}$ is the orbital angular velocity of the spacecraft rotating around the central gravitational body.

Infrared earth sensor model:

(12) $$\varphi_{h}=\varphi +b_{\varphi }+N_{\varphi h}+f_{\varphi }\;$$

(13) $$\theta_{h}=\theta +b_{\theta }+N_{\theta h}+f_{\theta }\;$$

where $\varphi_{h}$ is the ground sensitivity measurement output of the roll-axis, $b_{\varphi }$ is the ground sensitivity constant error of the roll-axis, $N_{\varphi h}$ is the ground sensitivity measurement noise of the roll-axis, $f_{\varphi }$ is the ground sensitivity fault of the roll-axis, $\theta_{h}$ is the ground sensitivity measurement output of the pitch-axis, $b_{\theta }$ is the ground sensitivity constant error of the pitch-axis, $N_{\theta h}$ is the ground sensitivity measurement noise of the pitch-axis, and $f_{\theta }$ is the ground-sensitive fault of the pitch axis.

Gyro measurement model:

(14) $$\left[\begin{array}{c} g_{1}\\ g_{2}\\ g_{3} \end{array}\right]=\left[\begin{array}{c} \omega_{x}\\ \omega_{y}\\ \omega_{z} \end{array}\right]+\left[\begin{array}{c} d_{1}+b_{1}\\ d_{2}+b_{2}\\ d_{3}+b_{3} \end{array}\right]+\left[\begin{array}{c} f_{g,1}\\ f_{g,2}\\ f_{g,3} \end{array}\right]$$

(15) $$g_{4}=\left[\begin{array}{ccc} \sqrt{3}/3 & \sqrt{3}/3 & \sqrt{3}/3 \end{array}\right]\left[\begin{array}{c} \omega_{x}\\ \omega_{y}\\ \omega_{z} \end{array}\right]+f_{g,4}$$

where $g_{i}$ is the gyro measurement output, $d_{i}$ is the gyro exponential drift term ($d_{i}=-\frac{1}{\tau_{i}}d_{i}$), $b_{i}$ is the gyro constant drift term, $f_{g,i}$ is the gyro fault, and *i* = 1, 2, 3.

Momentum wheel model:

(16) $$\;{\dot{h}}_{x}=J_{1}\dot{\bar{\omega_{1}}}=-u_{x}+f_{\bar{\omega },1}\;$$

(17) $${\dot{\;h}}_{y}=J_{2}\dot{\bar{\omega_{2}}}=-u_{y}+f_{\bar{\omega },2\;}$$

(18) $${\dot{h}}_{z}=J_{3}\dot{\bar{\omega_{3}}}=-u_{z}+f_{\bar{\omega },3\;\;}$$

where $J_{1}$, $J_{2}$, and $J_{3}$ are the rotary inertias of the momentum wheel; $\bar{\omega_{1}}$, $\bar{\omega_{2}},\;\;\mathrm{and}\;\bar{\omega_{3}}$ are the rotation speeds of the momentum wheel; $u_{x},u_{y},\;\mathrm{and}\;u_{z}$ are the expected output torques of the momentum wheel; $f_{\bar{\omega },1}$, $f_{\bar{\omega },2}$, and $f_{\bar{\omega },3}$ are the momentum wheel failures.

Based on the models of each part of the spacecraft control system, the entities and relationships of the performance-fault relationship diagram are extracted according to the following principles:

1. For each part of the spacecraft control system model, the known quantity and unknown quantity can be regarded as the “known quantity entity” and “unknown quantity entity”, respectively.
2. The purpose of this experiment is to infer whether there is a relationship among variables, so all kinds of errors, noises, constant parameters, and faults are not considered when constructing the graph.
3. When there are multiple variables on one side of the equation, an “*AND entity*” needs to be added to represent them.
4. The relationships of variables in the equation are divided into proportional equivalence, derivative equivalence, equivalence, addition, subtraction, and multiplication.

According to the above principles, the extracted entities are shown in Table 1.

**Figure 4:**
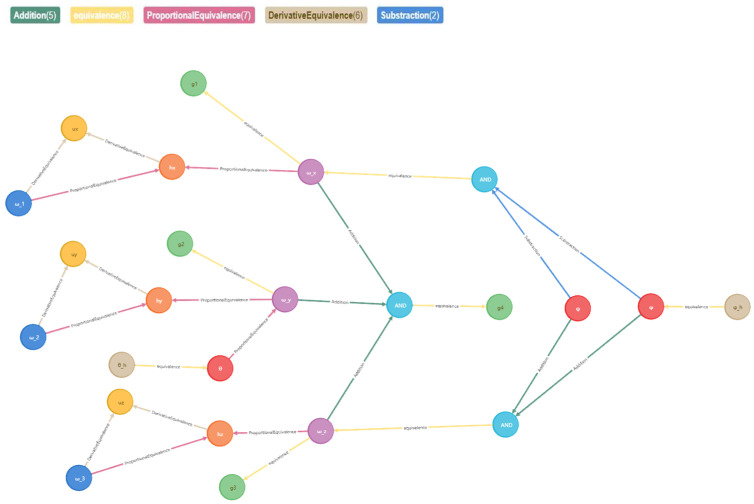
Performance-fault relationship graph of spacecraft control system.

According to the extracted entities and relationships, we construct the performance-fault relationship graph of the spacecraft control system, containing 24 entities, 5 relationships, and 28 triplets. As shown in Figure 4, the colors of connecting lines corresponding to different relationships are at the top.

### 3.2. Experimental Results of SPDDPG

**Figure 5:**
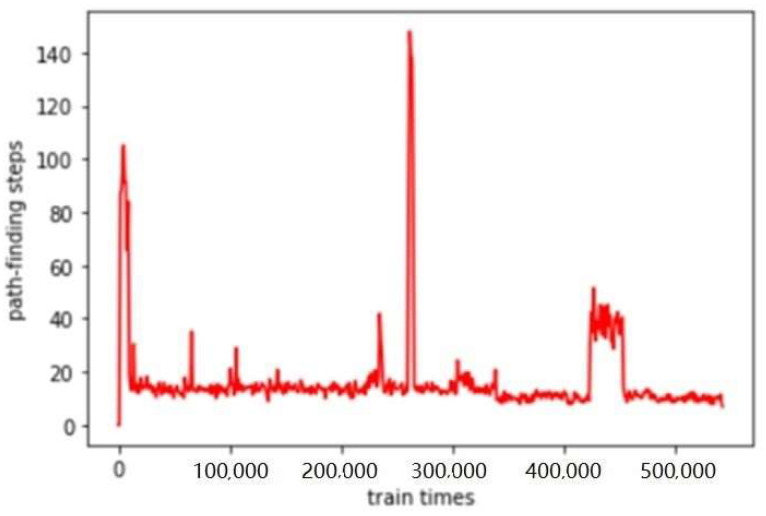
Average path-finding step learning curve.

**Figure 6:**
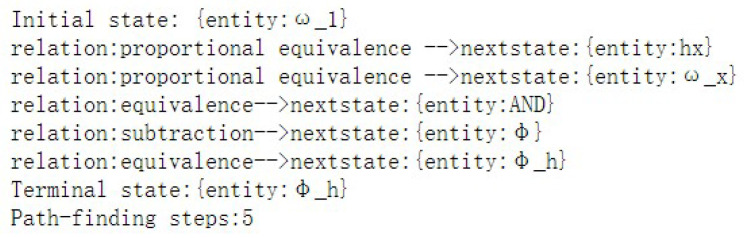
Visual output result of program.

**Figure 7:**
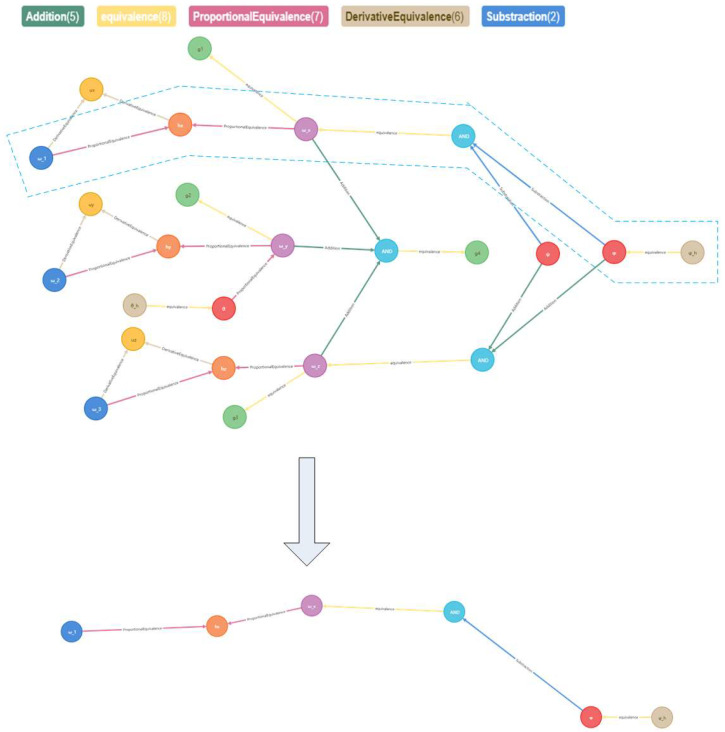
Visualization path finding process of Neo4j.

**Figure 8:**
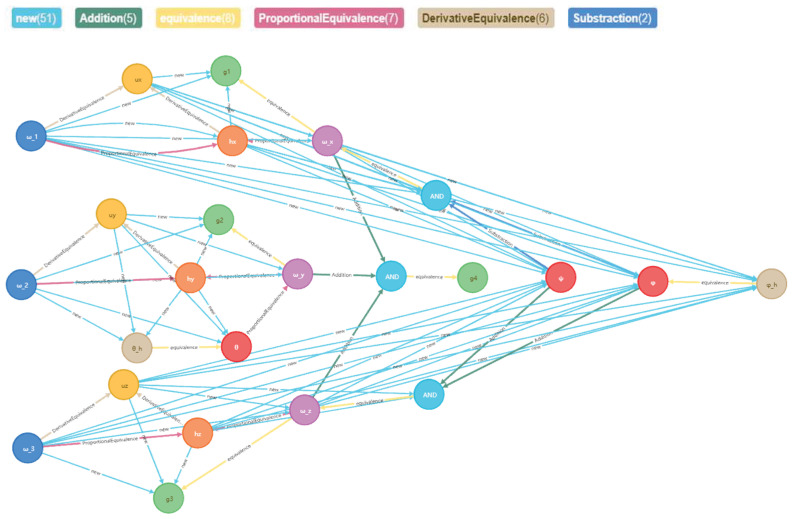
Completed performance-fault relationship graph of spacecraft control system.

We apply SPDDG to the performance-fault relationship graph of the spacecraft control system for relational reasoning and relational completion, and the parameters are set as follows. The output vector dimension of the TransE model is 100, and the dimension of the semantic vector after PCA dimension reduction is set to 20. The input of the actor network is a 42-dimensional state vector, and the output is a 7-dimensional predicted action vector. The number of neurons in the three hidden layers is set to (80, 80, 80), and the learning rate *lr* = 0.0005. The input of the critic network is a 40-dimensional vector, the output is a one-dimensional action value, the single hidden layer contains 60 neurons, and the learning rate *lr* = 0.0005. The training times are set to 60,000. Each training will randomly perform 100 pathfinding times. The longest relation path of the control system performance-fault relation graph is 7, so the training requirements can be met when the training times are reached or the average path-finding steps per hundred trainings are within seven steps. Actually, the training reaches the termination condition in 54,200 times, and the average path-finding step curve of the agent is as Figure 5:

We take the momentum wheel speed ω_1 as an example. It is taken as the initial state, and the infrared sensor output measurement *φ_h_* is taken as the final state. The path-finding step of the agent is 5, which costs 0.08 s, and the visual path-finding process is shown in Figure 6 and Figure 7:

The computer directly predicts the existence of the relationship between entities, and manually determines the path of entities that are difficult for the computer to directly determine. We use SPPDPG to discover 51 new relationships, and the completed spacecraft performance-fault relationship graph is shown in Figure 8.

### 3.3. Experimental Results of Various Models

To further verify the advantages of SPDDPG, we make a comparison between the performance of SPDDPG and other published models. Due to the practical application requirements, the relationship reasoning model applied to the spacecraft performance-fault relationship graph not only needs to predict the existence of the relationship between entities, but can also output the visual relationship path between entities for manual judgment. Therefore, we focus on selecting the reinforcement learning methods that meet the requirements, DeepPath [13] and MINERVA [15], and compare the experimental results from the path-finding accuracy and average path-finding steps. In order to verify the improvement of the DDPG algorithm, we add two DDPG models, DDPG(transE) and DDPG(State). DDPG(transE) only obtains the embedded vector of the entity and relationship through TransE, and DDPG(State) only sets the normal state and the termination state. The other parts of the two models are the same as SPDDPG. The experimental results are shown in Table 2, and the data are the best results obtained after many experiments.

The experimental results show that the SPDDPG algorithm has a good inference effect on the performance-fault relationship graph of the spacecraft control system. After the target entity is given, the accuracy of the agent finding the relationship path between entities reaches 100%, and the multi-hop relationship path between entities can be successfully obtained. In the performance-fault relationship graph, the longest relationship path is 7 steps, and the minimum average path-finding step number of agents is 6.91, which can control the path-finding step number to a few times and has high reasoning efficiency. In addition, compared with the experimental results of two kinds of DDPG models that have not been improved, two conclusions can be drawn: 1. The embedded vector extracted by semantic information and location information can contain more obvious entity features and improve the accuracy of relational reasoning. 2. Adding the imminent termination state to the normal state and the termination state can make the purpose of the agent’s routing clearer, reduce the path-finding steps, and improve the efficiency of relational reasoning. Therefore, this algorithm provides an interpretable reasoning means for completing the relationship of the spacecraft performance-fault relationship graph and has application significance.

## 4. Conclusions

The spacecraft performance-fault relationship graph has complex physical meanings, numerous entities, and coupling relationships; we propose a relationship reasoning model SPDDPG to solve the above problems, which combines the representation learning model and deep reinforcement learning model. Its advantages are as follows:

(1) The representation learning model is used to extract the semantic features of the entity and relationships, and the global location features of entities are obtained through Boolean information conversion. PCA is used to reduce the dimensions of entity vectors, to retain the high-order features of entities and avoid overfitting. It helps overcome the difficulty of distinguishing numerous entities, which is beneficial to the training of neural networks.
(2) The actor network is used to replace the traditional action selection strategy, and the critic network is used to fit the complex and uncertain value function. The deep neural network can distinguish complex physical meanings of the spacecraft performance-fault relationship graph and improve the efficiency of relationship reasoning.

We apply SPDDPG to the performance-fault relationship graph of the spacecraft control system, and the experimental results show that the algorithm has a good relationship reasoning effect on the knowledge graph of the spacecraft system level. In the future work, we are going to build a model of relationship prediction based on SPDDPG’s relationship reasoning path, so that it has both predictive ability and interpretability. We will apply this model to a larger spacecraft performance-fault relationship graph, and improve the reasoning efficiency.

## Figures and Tables

**Table 1:** Triple set of spacecraft control system model.

Known quantity entity	$\varphi_{h}$, $\theta_{h}$, $g_{1}$, $g_{2}$, $g_{3}$, $u_{x}$, $u_{y}$, $u_{z}$, $\bar{\omega_{1}}$*,*$\bar{\omega_{2}}$*,*$\bar{\omega_{3}}$, $h_{x}$, $h_{y}$, $h_{z}$, AND
Unknown quantity entity	$\omega_{x}$, $\omega_{y}$, $\omega_{z}$, $g_{4}$, φ, θ, ψ
Relation	equivalence, proportional equivalence, equivalence, derivative equivalence, addition, subtraction, multiplication
Triplet	$(g_{1}$, proportional equivalence, $\omega_{x}$); $(\omega_{x}$, addition, AND); $(\omega_{y}$, addition, AND); $(\omega_{z}$, addition, AND); $(g_{4}$, equivalence, AND)

**Table 2:** The comparison of experimental results.

Model	Path-Finding Accuracy	Average Path-Finding Steps
DeepPath	65.91%	19.43
MINERVA	72.86%	14.62
DDPG (transE)	77.64%	12.77
DDPG(State)	83.42%	14.29
SPDDPG	100%	6.91

## Data Availability

Not applicable.

## References

[1] Hogan A., Blomqvist E., Cochez M. (2021). Knowledge graphs. J. ACM Comput. Surv. (CSUR).

[2] Liu Q., Li Y., Duan H., Liu Y., Qin Z. (2016). Knowledge graph construction techniques. J. Comput. Res. Dev..

[3] Zhou Y.C., Wang W.J., Qiao Z.Y., Xiao M., Du Y. (2020). A survey on the construction methods and applications of scitech big data knowledge graph. Sci. Sin. Inf..

[4] Dong X., Gabrilovich E., Heitz G., Horn W., Lao N., Murphy K., Strohmann T., Sun S., Zhang W. Knowledge vault: A web-scale approach to probabilistic knowledge fusion. Proceedings of the 20th ACM SIGKDD International Conference on Knowledge Discovery and Data Mining.

[5] Nickel M., Murphy K., Tresp V., Gabrilovich E. (2015). A review of relational machine learning for knowledge graphs. Proc. IEEE.

[6] Wang Q., Mao Z., Wang B., Guo L. (2017). Knowledge Graph Embedding: A Survey of Approaches and Applications. IEEE Trans. Knowl. Data Eng..

[7] Lin Y., Han X., Xie R., Liu Z., Sun M. (2018). Knowledge representation learning: A quantitative review. arXiv.

[8] Paulheim H. (2016). Knowledge graph refinement: A survey of approaches and evaluation methods. Semant. Web.

[9] Wu T., Qi G., Li C., Wang M. (2018). A Survey of Techniques for Constructing Chinese Knowledge Graphs and Their Applications. Sustainability.

[10] Bordes A., Usunier N., Garcia-Duran A., Weston J., Yakhnenko O. (2013). Translating embeddings for modeling multi-relational data. Adv. Neural Inf. Process. Syst..

[11] Lao N., Mitchell T., Cohen W. Random walk inference and learning in a large scale knowledge base. Proceedings of the 2011 Conference on Empirical Methods in Natural Language Processing.

[12] Lao N., Minkov E., Cohen W. Learning relational features with backward random walks. Proceedings of the 53rd Annual Meeting of the Association for Computational Linguistics and the 7th International Joint Conference on Natural Language Processing (Volume 1: Long Papers).

[13] Xiong W., Hoang T., Wang W.Y. (2017). Deeppath: A reinforcement learning method for knowledge graph reasoning. arXiv.

[14] Wang H., Li S., Pan R., Mao M. Incorporating graph attention mechanism into knowledge graph reasoning based on deep reinforcement learning. Proceedings of the 2019 Conference on Empirical Methods in Natural Language Processing and the 9th International Joint Conference on Natural Language Processing (EMNLP-IJCNLP).

[15] Das R., Dhuliawala S., Zaheer M., Vilnis L., Durugkar I., Krishnamurthy A., Smola A., McCallum A. (2017). Go for a walk and arrive at the answer: Reasoning over paths in knowledge bases using reinforcement learning. arXiv.

[16] Shen Y., Chen J., Huang P.S., Guo Y., Gao J. (2018). M-walk: Learning to walk over graphs using monte carlo tree search. arXiv.

[17] Zeng X., He S., Liu K., Zhao J. Large scaled relation extraction with reinforcement learning. Proceedings of the Thirty-Second AAAI Conference on Artificial Intelligence.

[18] Lin X.V., Socher R., Xiong C. Multi-Hop Knowledge Graph Reasoning with Reward Shaping. Proceedings of the 2018 Conference on Empirical Methods in Natural Language Processing.

[19] Fu C., Chen T., Qu M., Jin W., Ren X. Collaborative Policy Learning for Open Knowledge Graph Reasoning. Proceedings of the 2019 Conference on Empirical Methods in Natural Language Processing and the 9th International Joint Conference on Natural Language Processing (EMNLP-IJCNLP).

[20] Bruna J., Zaremba W., Szlam A., LeCun Y. (2013). Spectral networks and locally connected networks on graphs. arXiv.

[21] Schlichtkrull M., Kipf T.N., Bloem P., Van Den Berg R., Titov I., Welling M. (2018). Modeling relational data with graph convolutional networks. European Semantic Web Conference.

[22] Vashishth S., Sanyal S., Nitin V., Talukdar P. (2019). Composition-based multi-relational graph convolutional networks. arXiv.

[23] Shang C., Tang Y., Huang J., Bi J., He X., Zhou B. End-to-end structure-aware convolutional networks for knowledge base completion. Proceedings of the AAAI Conference on Artificial Intelligence.

[24] Veličković P., Cucurull G., Casanova A., Romero A., Lio P., Bengio Y. (2017). Graph attention networks. arXiv.

[25] Nathani D., Chauhan J., Sharma C., Kaul M. Learning Attention-based Embeddings for Relation Prediction in Knowledge Graphs. Proceedings of the 57th Annual Meeting of the Association for Computational Linguistics.

[26] Xie Y., Zhang Y., Gong M., Tang Z., Han C. (2020). Mgat: Multi-view graph attention networks. Neural Netw..

[27] Chami I., Wolf A., Juan D.C., Sala F., Ravi S., Ré C. Low-Dimensional Hyperbolic Knowledge Graph Embeddings. Proceedings of the 58th Annual Meeting of the Association for Computational Linguistics.

[28] Wang D., Cui P., Zhu W. Structural deep network embedding. Proceedings of the 22nd ACM SIGKDD International Conference on Knowledge Discovery and Data Mining.

[29] Wang S., Wei X., Nogueira dos Santos C.N., Wang Z., Nallapati R., Arnold A., Xiang B., Yu P.S., Cruz I.F. Mixed-curvature multi-relational graph neural network for knowledge graph completion. Proceedings of the Web Conference 2021.

[30] Lillicrap T.P., Hunt J.J., Pritzel A., Heess N., Erez T., Tassa Y., Silver D., Wierstra D. (2015). Continuous control with deep reinforcement learning. arXiv.

[31] Abdi H., Williams L.J. (2010). Principal component analysis. Wiley Interdiscip. Rev. Comput. Stat..

